# MicroRNA-93 inhibits ischemia-reperfusion induced cardiomyocyte apoptosis by targeting PTEN

**DOI:** 10.18632/oncotarget.8941

**Published:** 2016-04-22

**Authors:** Zun-Ping Ke, Peng Xu, Yan Shi, Ai-Mei Gao

**Affiliations:** ^1^ Department of Cardiology, The Fifth People's Hospital of Shanghai, Fudan University, Shanghai, China; ^2^ Department of Emergency, The Affiliated Huai'an Hospital of Xuzhou Medical College and The Second People's Hospital of Huai'an, Huai'an, China; ^3^ Department of Pharmacy, The Fifth People's Hospital of Shanghai, Fudan University, Shanghai, China

**Keywords:** microRNA-93, ischemia/reperfusion, PTEN, AKT, apoptosis, Pathology Section

## Abstract

MicroRNAs have been implicated in some biological and pathological processes, including the myocardial ischemia/reperfusion (I/R) injury. Recent findings demonstrated that miR-93 might provide a potential cardioprotective effect on ischemic heart disease. This study was to investigate the role of miR-93 in I/R-induced cardiomyocyte injury and the potential mechanism. In this study, we found that hypoxia/reoxygenation (H/R) dramatically increased LDH release, MDA contents, ROS generation, and endoplasmic reticulum stress (ERS)-mediated cardiomyocyte apoptosis, which were attenuated by co-transfection with miR-93 mimic. Phosphatase and tensin homolog (PTEN) was identified as the target gene of miR-93. Furthermore, miR-93 mimic significantly increased p-Akt levels under H/R, which was partially released by LY294002. In addtion, Ad-miR-93 also attenuated myocardial I/R injury *in vivo*, manifested by reduced LDH and CK levels, infarct area and cell apoptosis. Taken together, our findings indicates that miR-93 could protect against I/R-induced cardiomyocyte apoptosis by inhibiting PI3K/AKT/PTEN signaling.

## INTRODUCTION

Myocardial ischemia/ reperfusion (I/R) injury contributes to adverse cardiovascular outcomes after myocardial ischemia, cardiac surgery or circulatory arrest, which represents a major cause of morbidity and mortality in humans with coronary heart disease. The molecular mechanisms underlying myocardial I/R injury, however, are complex, including oxidative stress, intracellular Ca^2+^ overload, rapid restoration of physiological pH upon reperfusion, the mitochondrial permeability transition pore, and exaggerated inflammation [1].

microRNAs (miRNAs) are a group of endogenous, non-coding, single-strand, small RNAs of 22-25 nucleotides, which downregulate the expression of multiple target genes *via* degradation or translational inhibition of their target mRNAs. Accumulating evidence [2, 3] has demonstrated that miRNAs have crucial roles in various cellular and biological processes, including cell growth, proliferation, differentiation, migration and apoptosis. Recently, it has been reported that miRNAs play an important role in myocardial I/R injury and have become important targets for therapeutic intervention. For example, knockdown of endogenous miR-320 provided protection against I/R-induced cardiomyocyte apoptosis by targeting heat shock protein 20 [4]. miR-7a/b was sensitive to I/R injury and protected myocardial cells against I/R-induced apoptosis by inhibiting poly (ADP-ribose) polymerase expression [5].

Increasing evidences also supports a pivotal role for miR-93 in multiple processes [6, 7], including tumorigenesis, metastasis, cell proliferation and apoptosis. However, the role of miR-93 in the cardiac I/R injury has rarely been reported. In the present study, we demonstrated that transfection with miR-93 mimic could attenuate myocardial injury and apoptosis induced by hypoxia/reoxygenation (H/R) in H9c2 cells. A bioinformatics analysis identified phosphatase and tensin homolog (PTEN) as an important candidate target for miR-93. Accordingly, we examined the possible involvement of PTEN in the protective action of miR-93.

## RESULTS

### miR-93 expression in H9c2 after H/R

**Figure 1:**
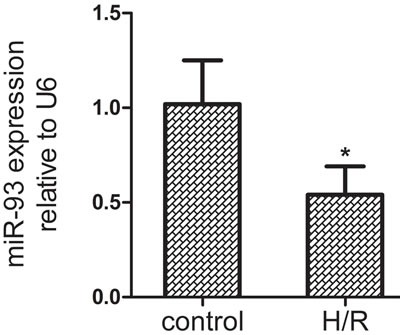
The expression of miR-93 in H9c2 cells by qRT-PCR after H/R treatment Data are expressed as mean ± SD (*n* = 3). *P < 0.05 *vs* the control group.

To identify the potential effect of miR-93 in myocardial I/R injury, we measured the expression of miR-93 in H9c2 cells after 10 h hypoxia and 2.5 h reoxygenation. As compared with controls, the expression of miR-93 was significantly down-regulated by 48% in H9c2 after H/R treatment (*P* < 0.05, Figure 1). This finding raised the possibility that miR-93 may play an important role in H/R injury of H9c2 cells.

### miR-93 ameliorates H/R-induced cardiomyocyte injury

**Figure 2:**
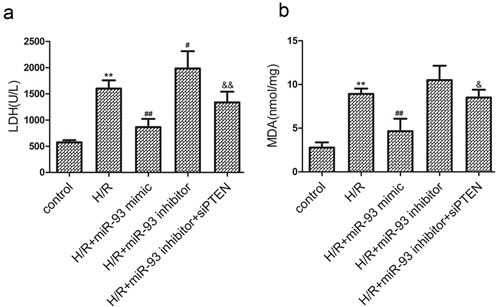
Effects of miR-93 on H/R-induced injury in H9c2 cells **a.** The level of LDH release. **b.** The level of MDA contents. Data are expressed as mean ± SD (*n* = 3). ^**^P < 0.01 *vs* the control group; ^#^*P* < 0.05, ^##^*P* < 0.01 *vs* the H/R group; ^&^*P* < 0.05, ^&&^*P* < 0.01 *vs* the H/R+ miR-93 inhibitor group.

LDH release is an indicator of cellular injury. Results from the experiment showed that H/R treatment increased LDH release in the culture media (Figure 2a). The miR-93 mimic significantly decreased LDH release, whereas the miR-93 inhibitor increased LDH release in response to H/R. In addition, co-transfection with siRNA-PTEN attenuated the effects of the miR-93 inhibitor.

MDA reflects cardiomyocyte oxidative damage. As shown in Figure 2b, the H/R-induced MDA release was significantly decreased by the miR-93 mimic. However, the miR-93 inhibitor increased MDA release in response to H/R, which was also attenuated by co-transfection with siRNA-PTEN.

### miR-93 decreases H/R-induced ROS production

**Figure 3:**
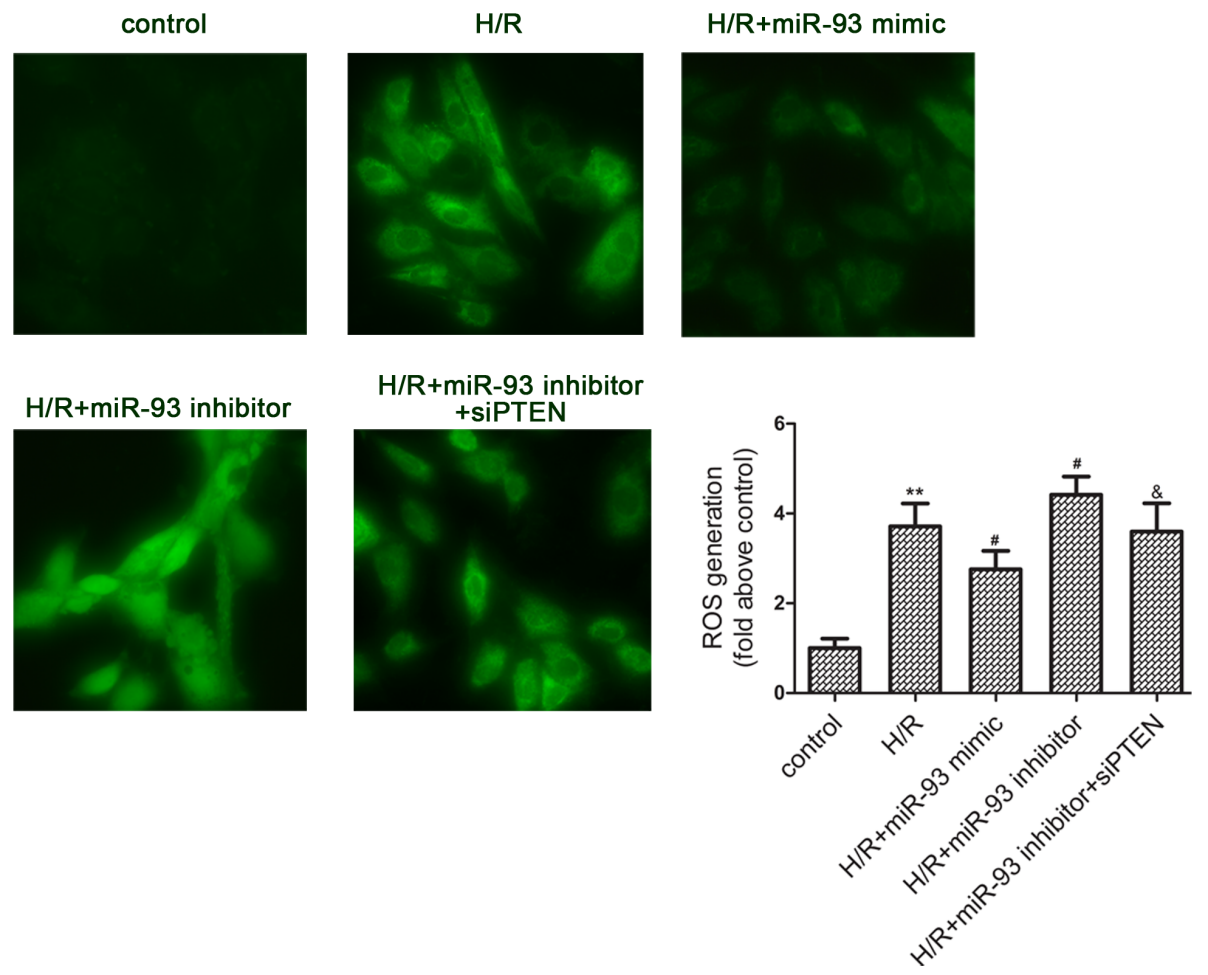
Effects of miR-93 on H/R-induced ROS generation in H9c2 cells Measurement of intracellular ROS by DCFH-DA staining (400× magnification). Data are expressed as mean ± SD (*n* = 3). ^**^P < 0.01 *vs* the control group; ^#^*P* < 0.05 *vs* the H/R group; ^&^*P* < 0.05 *vs* the H/R+ miR-93 inhibitor group.

Oxidative stress plays an important role in I/R induced cardiac injury. Therefore, we attempted to investigate the effect of miR-93 upon ROS generation. As shown in Figure 3, ROS levels were significantly increased by 177.15% by H/R treatment. Transfection with miR-93 mimic significantly decreased ROS generation to 66.67%, whereas transfection with miR-93 inhibitor increased ROS generation compared with H/R alone. However, co-transfection with siRNA-PTEN attenuated the effects of the miR-93 inhibitor on the intracellular concentration of ROS.

### miR-93 reduces H/R-induced cell apoptosis

**Figure 4:**
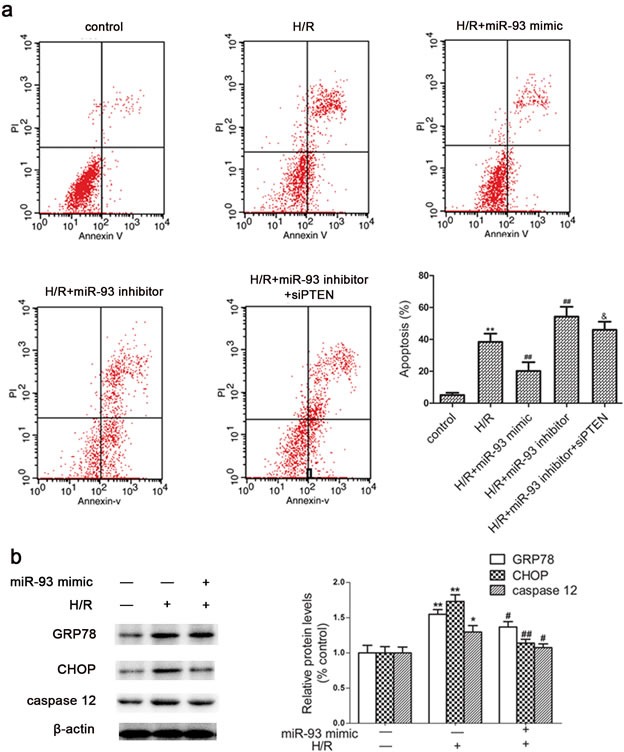
Effects of miR-93 on H/R-induced apoptosis in H9c2 cells **a.** Cells were stained with antibody to Annexin V-FITC and propiduim iodide after transfection with miR-93 mimic or inhibitor; Representative flow cytometry of apoptosis of cardiomyocyte under different conditions. **b.** Western blot analysis of protein level of GRP78, CHOP, and caspase-12 with miR-93 mimic transfected into H9c2 cells. Data are expressed as mean ± SD (*n* = 3). *P < 0.05, ^**^P < 0.01 *vs* the control group; ^#^*P* < 0.05, ^##^*P* < 0.01 *vs* the H/R group; ^&^*P* < 0.05 *vs* the H/R+ miR-93 inhibitor group.

Because miR-93 expression was inhibited by H/R in H9c2 cells, we wondered whether miR-93 protected cardiomyocyte against H/R-induced cell apoptosis. Flow cytometry revealed greater apoptosis with H/R than control treatment in H9c2 cells. Transfection with miR-93 mimic significantly decreased the apoptosis rate induced by H/R and Transfection with miR-93 inhibitor increased the apoptosis rate as compared with H/R alone (Figure 4). However, the effects of inhibitor were attenuated by cotransfection with siRNA-PTEN.

To further investigate the molecular mechanisms involved, the protein levels of endoplasmic reticulum stress (ERS) markers such as GRP78, CHOP, and caspase-12 were examined. As shown in Figure 4b, H/R treatment markedly increased the levels of GRP78, CHOP, and caspase-12, whereas presence of the miR-93 mimic significantly reverted these increased levels of GRP78, CHOP, and caspase-12.

### PTEN is a potential target of miR-93

**Figure 5:**
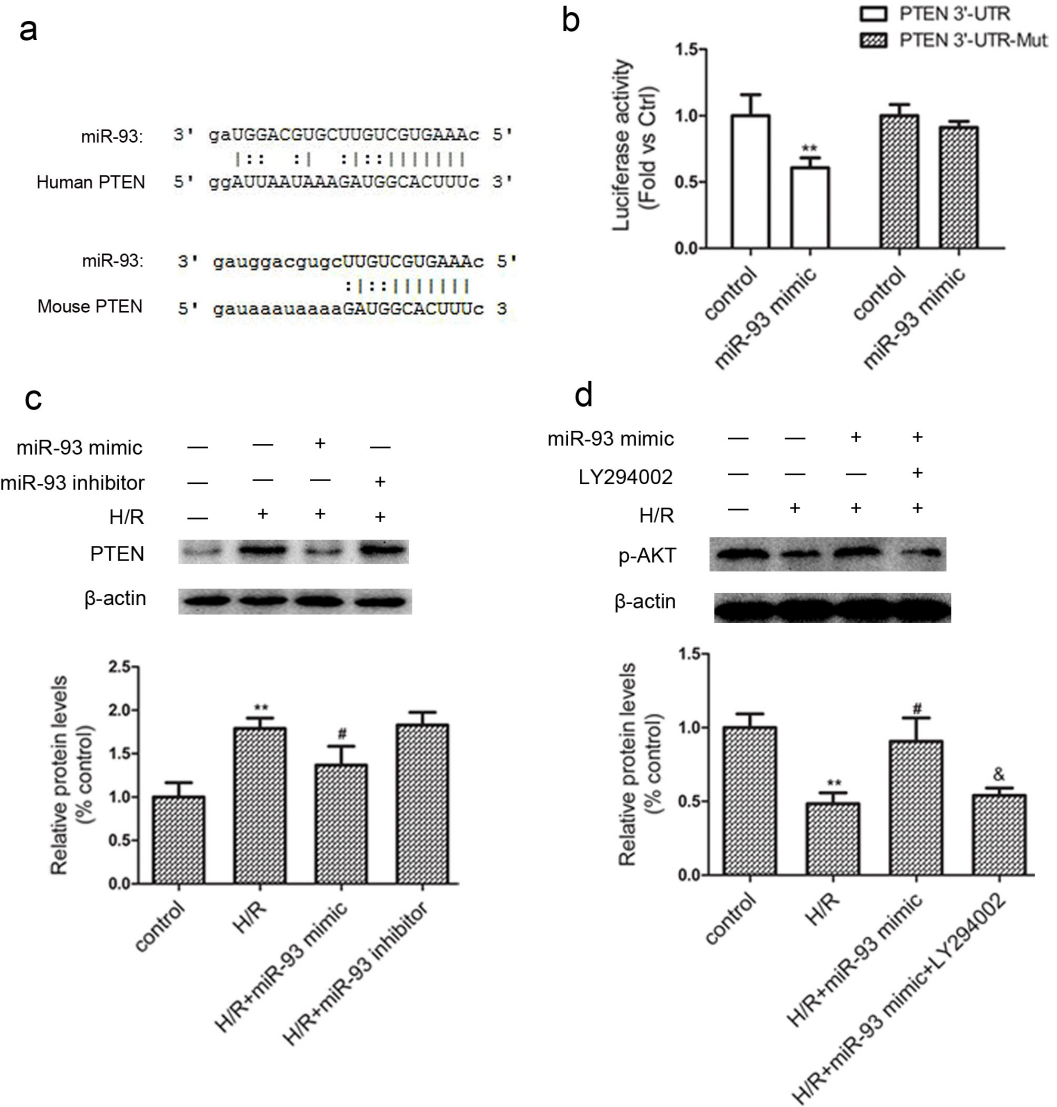
miR-93 targets PTEN **a.** The potential binding site for miR-93 in the 3ʹ-UTR of PTEN mRNA. **b.** Luciferase reporter assay was performed by co-transfection of 293T cells with luciferase reporter containing the 3ʹ-UTR of rat PTEN with miR-93 mimic. Luciferase activity was determined 24 h after transfection. **c.** Western blot analysis of protein level of PTEN with miR-93 mimic or inhibitor. **d.** Western blot analysis of protein level of p-AKT with miR-93 mimic or LY294002 (20 μM). Data are expressed as mean ± SD (*n* = 3). ^**^P < 0.01 *vs* the control group; ^#^*P* < 0.05 *vs* the H/R group; ^&^*P* < 0.05 *vs* the H/R+ miR-93 mimic group.

Bioinformatic analysis using ‘miRanda’ miRNA target prediction program revealed PTEN as one of the possible target gene of miR-93. Specifically, the 3′-UTR of the PTEN mRNA contains one binding site for miR-93 (Figure 5a). In comparison with the mutated control, the miR-93 mimic reduced the activity of the luciferase reporter fused with the PTEN 3′-UTR by 39% (Figure 5b). Western blot analysis revealed that the protein level of PTEN was increased by the miR-93 inhibitor, whereas the miR-93 mimic reduced the PTEN expression in response to H/R (Figure 5c). Furthermore, a significant inhibition of p-Akt levels in H9c2 after H/R treatment, and this inhibition was also ameliorated by miR-93 mimic (Figure 5d). However, 20μM of LY294002, a PI3K-Akt pathway inhibitor, could partially released p-Akt from miR-93 mimic-mediated elevation.

### miR-93 protects against myocardial I/R injury in rats

**Figure 6:**
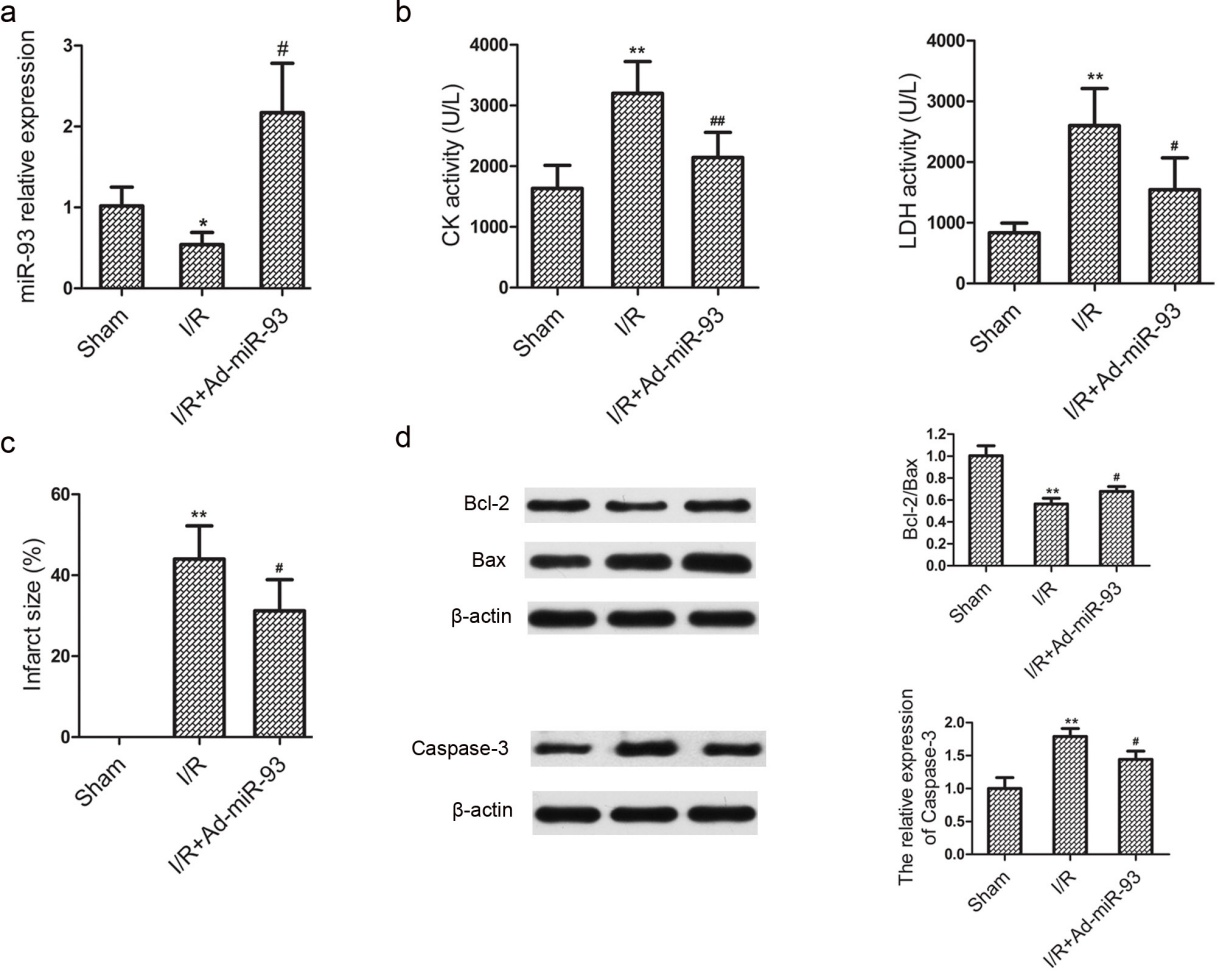
miR-93 protects against myocardial I/R injury *in vivo* **a.** Regulation of miR-93 after myocardial I/R injury. **b.** Effects of miR-93 on creatine kinase (CK) and lactate dehydrogenase (LDH) release after myocardial I/R injury. **c.** Effects of miR-93 on myocardial infarct size after myocardial I/R injury. d. Effect of miR-93 on apoptosis after myocardial I/R injury. ^*^P < 0.05, ^**^P < 0.01 *vs* the sham group; ^#^*P* < 0.05, ^##^*P* < 0.01 *vs* the I/R group.

As shown in Figure 6a, after myocardial ischemia for 30 min followed by 2 h of reperfusion, miR-93 expression was remarkably downregulated in rat hearts. Transfection of miR-93 into the myocardium could significantly increase miR-93 expression by 301.85 %. Furthermore, the activities of serum CK and LDH were significantly increased compared with sham group. However, the elevation in CK and LDH levels was obviously suppressed by Ad-miR-93 transfection (Figure 6b). Consistent with cardiac enzymes result, Ad-miR-93 transfection significantly reduced myocardial infarct size compared with that in the I/R group (Figure 6c). Additionally, myocardial cell apoptosis was also assessed. As shown in Figure 6d, the Bcl-2/Bax protein expression ratio was decreased in the I/R group, which were reverted in the I/R+Ad-miR-93 group. Moreover, the caspase-3 levels were upregulated in the I/R group, which were reverted in the I/R+ Ad-miR-93 group. Our findings strongly support that miR-93 also protects against myocardial I/R injury *in vivo*.

## DISCUSSION

Apoptosis plays a crucial role in myocardial I/R injury. A number of miRNAs [8–10], including miR-1, miR-15b, and miR-21, have been implicated in myocardial I/R injury due to their effects on key genes associated with apoptosis. Here, we investigated the role of miR-93 during myocardial I/R injury. *In vitro* studies, the expression of miR-93 was reduced by 48% after 10 h hypoxia and 2.5 h reoxygenation as compared with controls. Therefore, miR-93 is an I/R-related miRNA in cardiomyocyte.

Furthermore, we investigated the potential role of miR-93 in I/R-induced myocardial cell injury. We found that overexpression of miR-93 significantly decreased H/R-induced LDH release, MDA contents, ROS generation, and cell apoptosis *in vitro*. Signal transduction pathways involving GRP78, CHOP, and caspase-12 are known to mediate ERS-associated apoptosis [11–12]. Moreover, in this study we found the protein levels of GRP78, CHOP, and caspase-12 were increased by I/R injury, whereas miR-93 pretreatment reversed these effects. Therefore, overexpression of miR-93 may be responsible for decreasing ERS-associated apoptosis and protecting cardiomyocyte during I/R injury. *In vivo* study, we also found that Ad-miR-93 could attenuate myocardial I/R injury, manifested by reduced LDH and CK levels, infarct area and cell apoptosis in rats.

MiRNAs enforce their function *via* degradation or translational inhibition of their target mRNAs at the post-transcriptional level [13]. PTEN, a tumor suppressor gene, which was an essential regulator of cell proliferation, apoptosis, differentiation, migration, etc [14]. Recently, studies have found that PTEN plays an important role in myocardial remodeling, cardiac hypertrophy, myocardial fibrosis and myocardial ischemia reperfusion injury [15–17]. Based on bioinformatic analyses, PTEN was identified as a target of miR-93. Such a prediction was confirmed by a dual luciferase reporter assay. Therefore, PTEN is a functional target gene of miR-93 involved in cardiomyocyte protection against I/R injury.

PI3K/Akt is an intracellular signaling pathway, which also involve in the cardioprotection [18]. PI3K, when activated, has the ability to phosphorylate PIP2 into the secondary messenger PIP3 and lead the activation of Akt. When Akt is activated, it may product its antiapoptotic effects *via* the phosphorylation of two categories of downstream substrates: the antiapoptotic substrates (such as: Bcl-2, eNOs, p70s6k) and the proapoptotic substrates (such as bad, Bax, caspase9, GSK-3β) [19]. Moreover, PTEN is the main negative regulator of PI3K/Akt pathway [20]. So, our results showed that miR-93 can signal through a PTEN/Akt axis in the cardiomyocytes, which inhibits cardiomyocytes apoptosis during I/R injury.

This study provided evidence for the first time that miR-93 can decrease cardiomyocyte apoptosis induced by I/R *via* inhibiting PTEN and increasing nuclear AKT. PTEN may be the target gene of miR-93 against I/R-mediated cardiomyocyte apoptosis. miR-93 may be a potential drug target for treating cardiomyocyte I/R injury. Further research in human clinical trials are necessary to pave the way for miR-93 ultimate application in the clinic to benefit myocardial I/R patients.

## MATERIALS AND METHODS

### Materials and reagents

MiR-93 mimic, inhibitor and matched negative control (NC) were synthesized by GenePharma, Shanghai, China. Lactate dehydrogenase (LDH), creatine kinase (CK) and malondialdehyde (MDA) commercial kits were purchased from Nanjing Jiancheng Bioengineering Institute (Nanjing, China). Reactive oxygen species (ROS) kit, Annexin V-FITC/propidium iodide (PI) apoptosis kit, and LY294002 were purchased from Beyotime Institute of Biotechnology (Shanghai, China). PTEN, glucose-regulated protein (GRP) 78, C/EBP homologous protein (CHOP), caspase-12, P-AKT, Bax, Bcl-2, caspase-3 and β-actin antibodies were obtained from Santa Cruz Biotechnology (USA). HiPerFect Transfection reagent and miRNeasy Mini kits were from Qiagen (Germany). Quick-change Mutagenesis Kit were from Stratagene (Germany). A dual luciferase psiCheck-2 reporter plasmid and dual luciferase reporter assay kit were from Promega (USA).

### Cell culture

H9c2 cells, which derived from Cell Bank of the Chinese Academy of Sciences, were cultured in DMEM containing 10% fetal bovine serum in a humidified incubator containing 5% CO_2_ at 37°C [5].

### miRNA transfection

H9c2 cells were seeded into six-well plates and transfected with miR-93 mimics, miR-93 inhibitor, or miR-NC using HiPerFect Transfection reagent according to the manufacturer's instructions. Subsequent experiments including H/R, RNA/protein extraction and apoptosis analysis were performed 24 h after miRNA transfection.

### *in vitro* I/R model

Briefly, cardiomyocytes were firstly perfused in normal Hank's solution with a gas mixture of 95% O2-5% CO2 at 37°C, pH 7.4. To simulate ischemia, the Hank's solution was switched to pH 7.4 at 37°C without glucose or calcium and then the cells were aerated with a gas mixture of 95% N2-5% CO_2_ for 10 h. To simulate reperfusion, the cells were again treated with normal Hank's solution with a gas mixture of 95% O2-5% CO2 at 37°C, pH 7.4 for 2.5 h [8]. Cells under normoxia throughout the experiments were included as a control.

### *In vivo* gene transfer and rat myocardial I/R model

Sprague-Dawley rats (250-300 g) were anesthetized with pentobarbital, and the hearts were exposed. Then the Ad-miR-93 was respectively injected into the left ventricular anterior wall. The chest was closed after injection and the rat was allowed to recover. Myocardial I/R treatment was performed 4 days later. All the rats were re-anesthetized. The heart was exposed and the left anterior descending coronary artery (LAD) was ligated using silk suture for 30 min. Myocardial ischemia was confirmed by myocardial blanching and electrocardiography evidence of injury. Subsequent to that, the LAD was reperfused for 2 h. Then, the hearts and blood samples were obtained for further analysis. In the sham group, the heart was exposed without ligating the LAD. All animal experiments were approved by the animal ethics committee of the Fifth People's Hospital of Shanghai, Fudan University.

### Real-time quantitative PCR (qRT-PCR)

MicroRNAs were isolated from cultured cells with a miRNeasy Mini Kit. The primers were as follows: pri-U6: 5ʹ-CTCGCTTCGGCAGCACA-3ʹ and 5ʹ-AACGCTTCACGAATTTGCGT-3ʹ; pri-miR-93: 5ʹ-AAGTGCTGTTCGTGCAGGT-3ʹ and 5ʹ-CTCGGGAAGTGCTAGCTCA-3ʹ. qPCR was performed on a Rotor-Gene 3,000 real-time DNA detection system using the TaqMan MicroRNA Assay kit in accordance with the manufacturer's instructions. Gene expression was determined by comparing the data against the standard curve, and normalized against U6.

### Detection of LDH, CK and MDA

Three specific marker enzymes, including the LDH, CK and MDA were measured using commercial kits according to manufacturer's instructions.

### Intracellular ROS assay

H9c2 cells were incubated with 10 μM 2ʹ, 7ʹ-dichlorofluorescin-diacetate at 37°C for 30 min in the dark. Then, the plates were washed three times with phosphate-buffered saline. The DCFH-DA stain detecting ROS production was observed using a fluorescence microscope (Nikon, Japan).

### Apoptosis analysis

The Annexin V-FITC/PI apoptosis detection kit was used to determine the cell apoptosis, according to the manufacturer's instructions. After transfection, cells were harvested and resuspended in 200 μl binding buffer. Then, the cells were incubated with 10 μl Annexin V-FITC and 5 μl PI in the dark for 15 min. The apoptosis rate was evaluated by flow cytometric analysis.

### Luciferase reporter assay

A dual luciferase psiCheck-2 reporter plasmid was used to harbored the 3′-UTR of the PTEN. Site-directed mutagenesis of the miR-93 target-site in the PTEN-3'-UTR was performed using the Quick-change Mutagenesis Kit and named PTEN 3′-UTR-mut. For the mutated construct, the miR-93 target site AAAGTGC was substituted with a TTTCACG fragment. Transfection was performed in triplicate with miR-93 mimic and PTEN 3′-UTR-wild or PTEN 3′-UTR-mut. Co-transfection with non-targeting negative control RNA was performed as a control. The cells were harvested 24 h after transfection for luciferase activity using a dual luciferase reporter assay kit on a luminometer [5].

### Western blotting

Samples were separated on SDS-polyacrylamide gels and transferred to PVDF membranes. For immunoblotting, PVDF membranes were blocked with 5% skim milk at room temperature for 2 h and probed with antibodies overnight at 4°C overnight followed by incubation with secondary antibody conjugated to horseradish peroxidase for 1.5 h. Bands were visualized with an enhanced chemiluminescence detection kit following the manufacturer's instructions.

### Determination of infarct size

After reperfusion, 3 mL 2% (w/v) Evans blue was injected into to the left anterior descending coronary artery in order to visualize ischemic and non-ischemic areas. After washing, cooling, and sectioning, the left ventricle slice was stained with 1% TTC (pH 7.4) at 37°C for 15 min. TTC did not stain the infarcted myocardium, thus showing white in color while nonischemic myocardium was stained by TTC and showed brick-red in color. Results were analyzed for the infarcted area using the Image J software and expressed as the percentage of left ventricular volume for each heart.

### Statistics analysis

Experiments were performed in triplicate and data were expressed as mean ± standard deviation (SD). Statistical significance was determined by one-way analysis of variance (ANOVA). A value of *P* < 0.05 was considered statistically significant.
